# Characterization of Mineral and Synthetic Base Oils by Gas Chromatography–Mass Spectrometry and Fourier Transform Ion Cyclotron Resonance Mass Spectrometry

**DOI:** 10.1021/acs.energyfuels.2c02437

**Published:** 2022-11-02

**Authors:** Shinjong Lee, Diana Catalina Palacio Lozano, Hugh E. Jones, Kyongsik Shin, Mark P. Barrow

**Affiliations:** † Materials Technology and Analysis Team, Hyundai Motor Group, Gyeonggi-do18280, Korea; ‡ Department of Chemistry, University of Warwick, CoventryCV4 7AL, United Kingdom

## Abstract

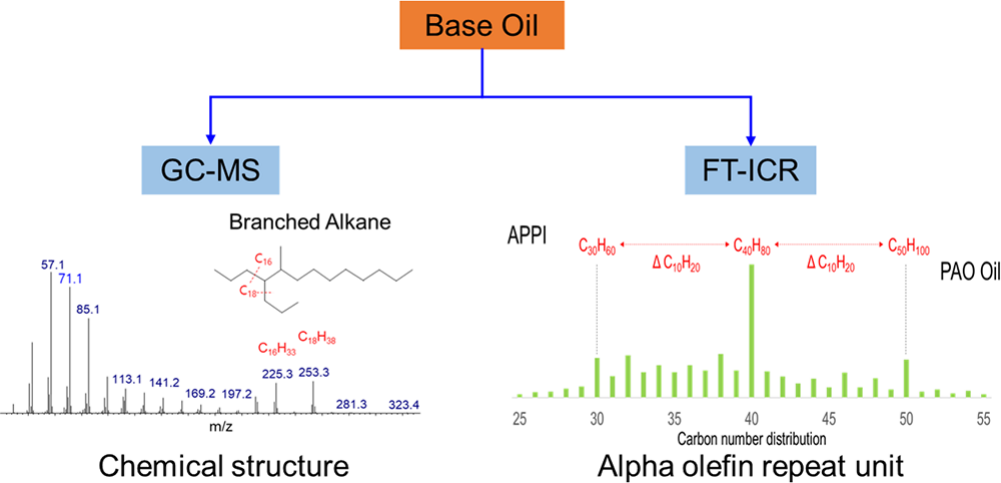

Base oil is a main component of engine oil that enables smooth operation of an internal combustion engine. There are two types of base oils, such as mineral oil and synthetic oil. In this study, Fourier transform ion cyclotron resonance mass spectrometry (FT-ICR MS) and gas chromatography–mass spectrometry (GC–MS) were used to characterize the base oils. One difficulty in analyzing base oils using MS is that the ionization of alkanes can be problematic due to low ionization efficiencies and the predominance of fragmentation. Despite these limitations, the combination of GC–MS and FT-ICR MS data can provide qualitative insights into the composition differences for these various sample types. The distinctive total ion chromatogram obtained by GC–MS of the different base oils allowed the classification of mineral oil from synthetic oil. The additional structural characteristics of paraffinic compounds were also inferred by GC–MS. FT-ICR MS coupled to two different ionization methods, atmospheric pressure photoionization (APPI) and atmospheric pressure chemical ionization (APCI), was tested for the analysis of base oils. It was determined that APPI was suitable for the analysis of aliphatic hydrocarbon compounds, where APPI minimizes the decomposition of hydrocarbon compounds compared to atmospheric pressure chemical ionization. Using APPI FT-ICR MS, the components of the oils were characterized, including not only paraffinic compounds but also cyclic compounds. In addition, the alpha olefin monomer of the synthetic oil was determined, and the homogeneity of the branched compound of the synthetic base oil was confirmed using GC–MS and FT-ICR MS results.

## 1 Introduction

An internal combustion engine vehicle operates using the energy generated when fuel combusts at high temperatures inside the engine. As the engine transmits energy through fast rotational motion, the role of engine oil is crucial for the smooth movement of the motor. Engine oils are constituted of a base oil, a viscosity modifier, and functional additives. The base oil properties may vary depending on their molecular compositions, and therefore, the selection of additives and viscosity modifiers, as well as the choice of the base oil, needs to be tuned to meet the performance subject to the application.

Base oils are classified into two main groups: mineral oils which are refined from petroleum-based hydrocarbons and synthetic base oils corresponding to hydrocarbons derived from pure chemical reactions. The American Petroleum Institute (API) has categorized base oils into five groups, as shown in Table 1.

**Table 1:** Properties of API-Defined Base Oil Groups^1^

API group	saturate level (%)	sulfur level (%)	viscosity index
Group I	<90	>0.03	80–120
Group II	≥90	≤0.03	80–120
Group III	≥90	≤0.03	≥120
Group IV	100% PAOs (poly-alpha-olefins)		
Group V	all others not included in Groups I–IV		

Group I, II, and III base oils are categorized as mineral oils. Group II and III oils are majority saturated (≥90%), with a higher percentage of normal-, iso-, and cyclo-paraffin (naphthenes) than solvent-refined (Group I) oils.^2^ The category Group II+ also exists as an unofficial term unrecognized by API, where these oils have a higher viscosity index than standard Group II oils of approximately 115, and typically have been produced through hydrotreatment.

Group IV and V base oils are categorized as synthetic oils, where Group IV comprises poly(α-olefins) (PAO) and Group V corresponds to oils not defined in the previous groups. Group IV PAOs tend to be a mixture of pure branched alkane hydrocarbons and are produced for applications where a high temperature stability is required.^3^ Some engine oils use synthetic oil mixed with mineral oils, as synthetic oils tend to be considerably more expensive than mineral oils. The ratio of mineral oils to synthetic oils can clearly affect the engine performance, and, therefore, it is important to understand and characterize the main compositional differences between the different types of base oils.

Base oils are complex compositions often containing a mixture of volatile and nonvolatile chemicals, and, therefore, a combination of sophisticated techniques is often needed to get greater insights into their chemical composition.^2,4−7^ Hourani et al.,^8^ performed the analysis of Group I and Group III base oils using different analytical methods, such as FT-ICR MS, two dimensional gas chromatography (2D-GC), and high-performance liquid chromatography (HPLC). In general, the base oil samples mostly comprised paraffinic and naphthenic structures, alongside mono-, di-, and tri(+)-aromatic hydrocarbons. According to their results, base oils classified as Group I contain a higher percentage of aromatic molecules, while Group III samples contain mostly saturated species. Scheuremann et al.^9^ analyzed different PAOs corresponding to Group IV by using GC–MS. In this work, the large number of isomers of the PAO oligomer C_20_H_42_, were clearly identified. To overcome the low resolution of GC–MS, a long capillary column (length: 150 m) and long analysis time (620 min) were necessary. However, due to the diverse isomeric compositions of the PAOs, the overall molecular differences were difficult to be explained. Giri et al.^10^ used electron ionization (EI) and photoionization (PI) coupled to GC × GC-ToF (time-of-flight) MS to analyze lubricant oil samples. Due to the lack of mass spectral libraries of standard material that can be used to assign molecular structures, a range of mass spectra were acquired with variable electron energy (10–70 eV). The overall analyses performed to date, therefore, address the characterization of either mineral oils or base oils using a combination of GC–MS and high-to-ultrahigh-resolution MS.

In this study, the characterization of five base oils corresponding to Group II, II+, III, and two Group IV base oils with different viscosity indices was analyzed using GC–MS and FT-ICR MS coupled to atmospheric pressure photoionization (APPI) and atmospheric pressure chemical ionization (APCI).

## 2 Experimental Section

### 2.1 Chemicals and Samples

Base oils from Group II, II+, III, and two base oils categorized as Group IV were analyzed in this study. The properties of each sample can be found in Table 2. Mineral base oil sample A corresponds to the base oils of Group II (G II); sample B corresponds to Group II+ (G II+); and sample C belongs to Group III (G III). Synthetic base oil samples D and E belong to base oil Group IV (G IV). Analytical grade solvents were purchased from Sigma-Aldrich, unless specified otherwise.

**Table 2:** Sample Information of Base Oil

no	API definition	sample name	viscosity index	type of base oil
1	Group II	A	107	mineral oil
2	Group II+	B	116	
3	Group III	C	123	
4	Group IV	D	124	synthetic oil
5	Group IV	E	143	

### 2.2 GC–MS Analysis

### 2.3 FT-ICR MS Analysis

FT-ICR MS analyses were performed at the University of Warwick using a Bruker solariX equipped with a 12 T superconducting magnet (Bruker Daltonik GmbH, Bremen, Germany) coupled to an APCI source or an APPI ionization source, each operated in positive-ion mode. The APPI source was equipped with a krypton lamp which emits photons at 10.0 and 10.6 eV. The samples were diluted to 10 μg/mL in *n*-hexane for APCI experiments and to 10 μg/mL in dichloromethane for APPI experiments. The resulting sample solutions were analyzed by direct infusion FT-ICR MS experiments without the addition of a base or further treatment. All solvents used were analytical grade reagents. Glassware was used for all solvent handling and transfer, except for the pipette tips used with micropipettes. The sample solution was injected into the ionization source at 500 μL/h using a syringe pump. Nitrogen gas was passed through the heated nebulizer and used as the drying gas, where the heated nebulizer temperature was set to 260 °C, and the drying gas was maintained at 180 °C. The corona needle current of the APCI source was set to 2100 nA, and the capillary potential was 4500 V. A total of 280 scans were accumulated to improve the signal-to-noise ratio of the resulting spectra. Data sets of 4 MW with a resolution of 180,000 at *m/z* 400 were acquired, covering the range *m/z* 100–1500.

### 2.4 Data Analysis

FT-ICR MS spectra were externally calibrated using “ESI Tuning Mix” (Agilent Technologies, Milton Keynes, UK), followed by an internal recalibration with a series of aliphatic hydrocarbons using Data Analysis 5.0 (Bruker Daltonik GmbH, Bremen, Germany). The *m/z* values between 100 and 700 were used with relative abundances greater than 6 times the standard deviation of the baseline noise. Composer 1.5.6 (Sierra Analytics Inc., Modesto, CA, USA) was used to assign the elemental molecular compositions in each mass spectrum. The compositions were assigned with the following constraints: C_4–200_, H_4–1000_, S_0–2_, O_0–6_, and N_0–2_, with a mass error of <1 ppm. The number of double-bond equivalents (DBE), or number of rings plus double bonds to carbon, was calculated according to the following equation:
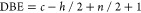
1where *c*, *h*, and *n* are the number of carbon, hydrogen, and nitrogen atoms, respectively. Each individual molecular assignment can be categorized by its heteroatomic class *S*_s_*O*_o_*N*_n_, carbon number (number of carbon atoms within the molecule), and DBE. Further data analysis and visualization were performed using the in-house tool, KairosMS.^11^

## 3 Results and Discussion

### 3.1 Identification of Hydrocarbons by GC–MS

**Figure 1:**
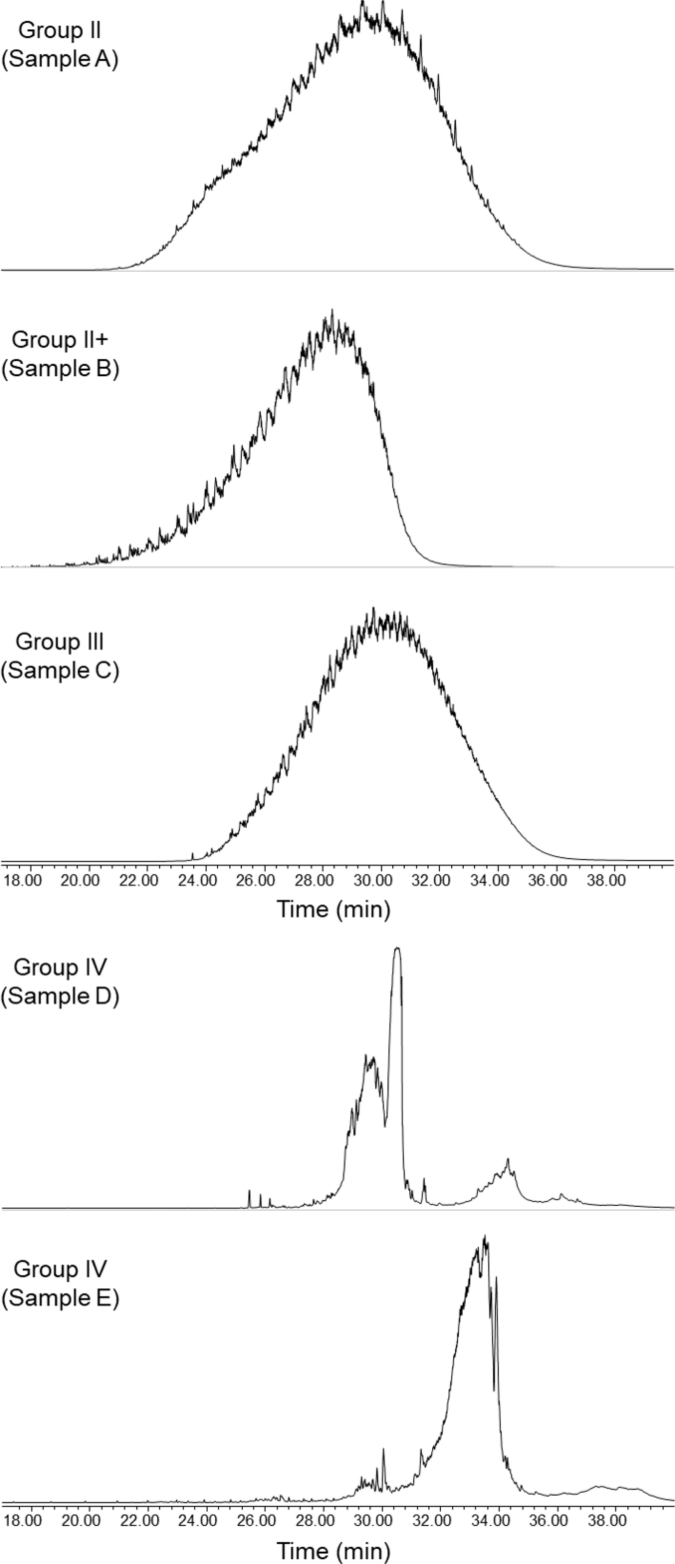
Total ion chromatograms of base oils by GC–MS.

**Figure 2:**
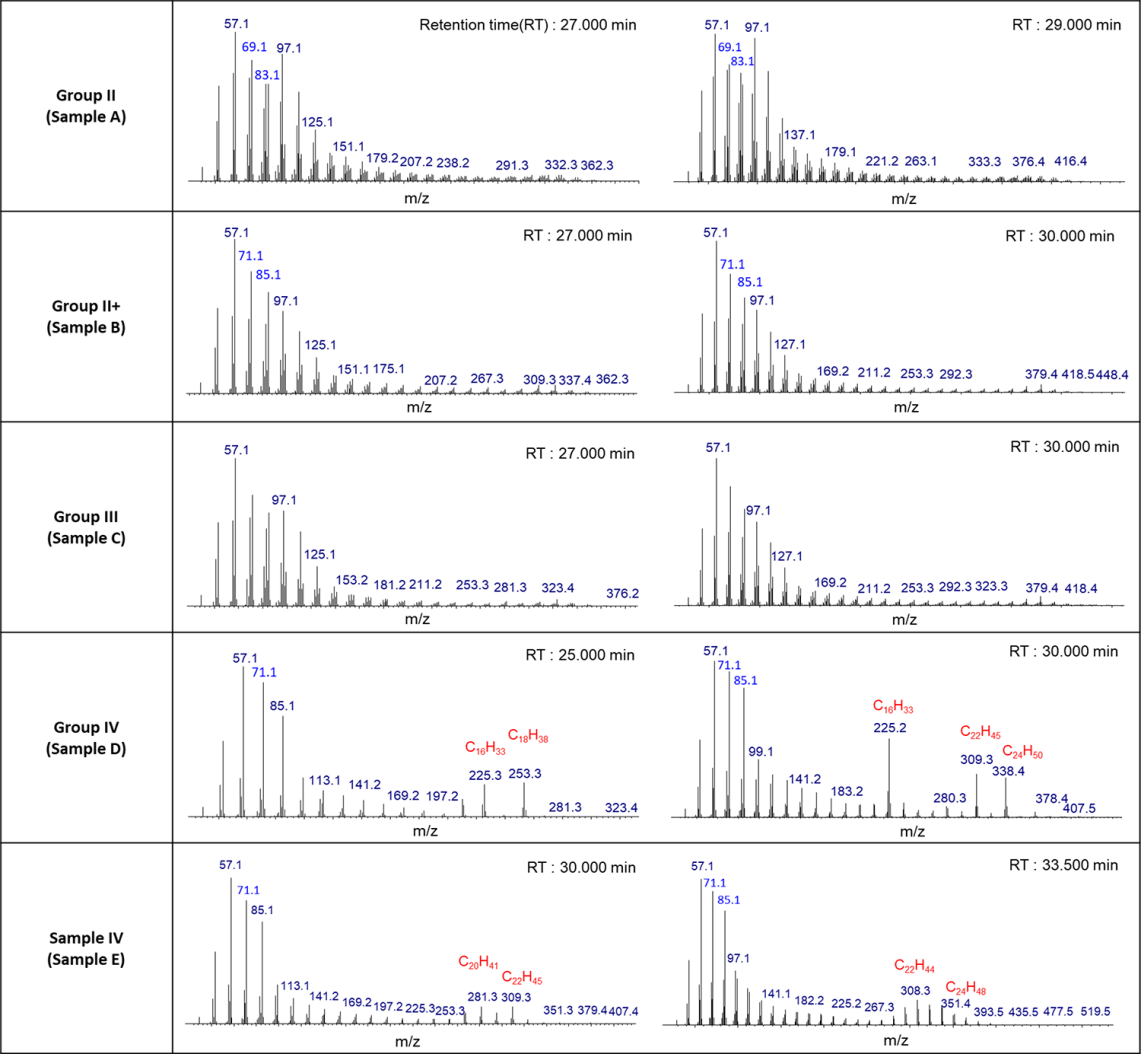
Mass spectra of base oils obtained by GC–MS.

Five base oils were analyzed using GC–MS. The chromatograms can be found in Figure 1, and examples of associated mass spectra, including examples of compositional assignments, can be found in Figure 2.

As can be seen in Figure 1, three types of base oils classified as Group II/Group II+/Group III, the so-called mineral oils, showed hump-type chromatograms in which components were continuously detected. This means that the mineral oils are composed of compounds with similar molecular weights and that the samples were sufficiently complex that they could not be separated by retention time alone. On the other hand, two types of base oils classified as Group IV, the so-called synthetic oils, showed discontinuous chromatograms, with narrower distributions. This indicates that the synthetic oils are composed of compounds with more specific molecular weight ranges than the mineral oils.

The shape of each chromatogram is very distinctive for each base oil. Mineral base oils showed a continuous elution of components spanning a wide range of retention times on the column. It is noticeable that Group II+ compositions eluted from the column at a lower retention time, which indicates compositions with a lower molecular weight in comparison with mineral oils from Groups II and III. In a similar way, it was possible to observe important differences between the synthetic oils D and E. As can be seen in Figure 1, the majority of compositions from sample D eluted from the column between 28 and 30 min of the GC–MS analysis, whereas the compositions of sample E eluted at 32–34 min. This implies compositions with a higher molecular weight, which would be in line with the higher viscosity of sample E.

As a consequence of the low chromatographic resolution of traditional GC–MS, it is not possible to separate the individual components of the base oil; however, important differences in the chemical structures of the components can be inferred from the mass spectra. Figure 2 shows the fragmentation mass spectra of the base oils. Mass spectra were acquired at two different retention times for each sample: at retention times of 27 and 29 min for Group II, 27 and 30 min for Group II+, 27 and 30 min for Group III, 25 and 30 min for Group IV (sample D), and 30 and 33.5 min for Group IV (sample E).

The fragments detected in Group II/II+/III (samples A/B/C) base oils show the typical distribution of linear alkane fragmentation, that is, a wide distribution with fragments distributed at low *m/z* values. In general, the fragment detected at *m/z* 57.1 was observed in all mineral oils. Group II (sample A) only shows *m/z* 83 and *m*/*z* 69 which are the indicators of alkene and cycloalkane, but Group II+/III (samples B/C) show *m/z* 85 and *m/z* 71. All mineral oils show *m/z* 57 and *m/z* 43. This indicates that the main components of samples A/B/C are linear alkane. On the other hand, Group IV (samples D/E) base oils showed a strong fragment at around *m/z* 57 and a medium-intensity fragment (second hump at higher *m/z*) at *m/z* 225.3 (for sample D) and *m/z* 337.4 (for sample E). The fragment at *m/z* 225.3 suggests a C_16_H_33_^+^ alkane fragment (theoretical: *m/z* 225.257676), with the fragment at *m/z* 337.4 corresponding to C_24_H_49_^+^ (theoretical: *m/z* 337.382876), and the possible chemical structures for these compositions have been proposed by Sebastian and co-workers^9^ and Giri et al.^12^ The fragment of the second hump in the mass spectrum is likely due to cleavage at the branched position and therefore can be characteristic of the isomeric species present at that retention time. The chemical formula of an alkyl branch can be estimated from the molecular weight.

### 3.2 Identification of Hydrocarbon Compounds by FT-ICR MS

**Figure 3:**
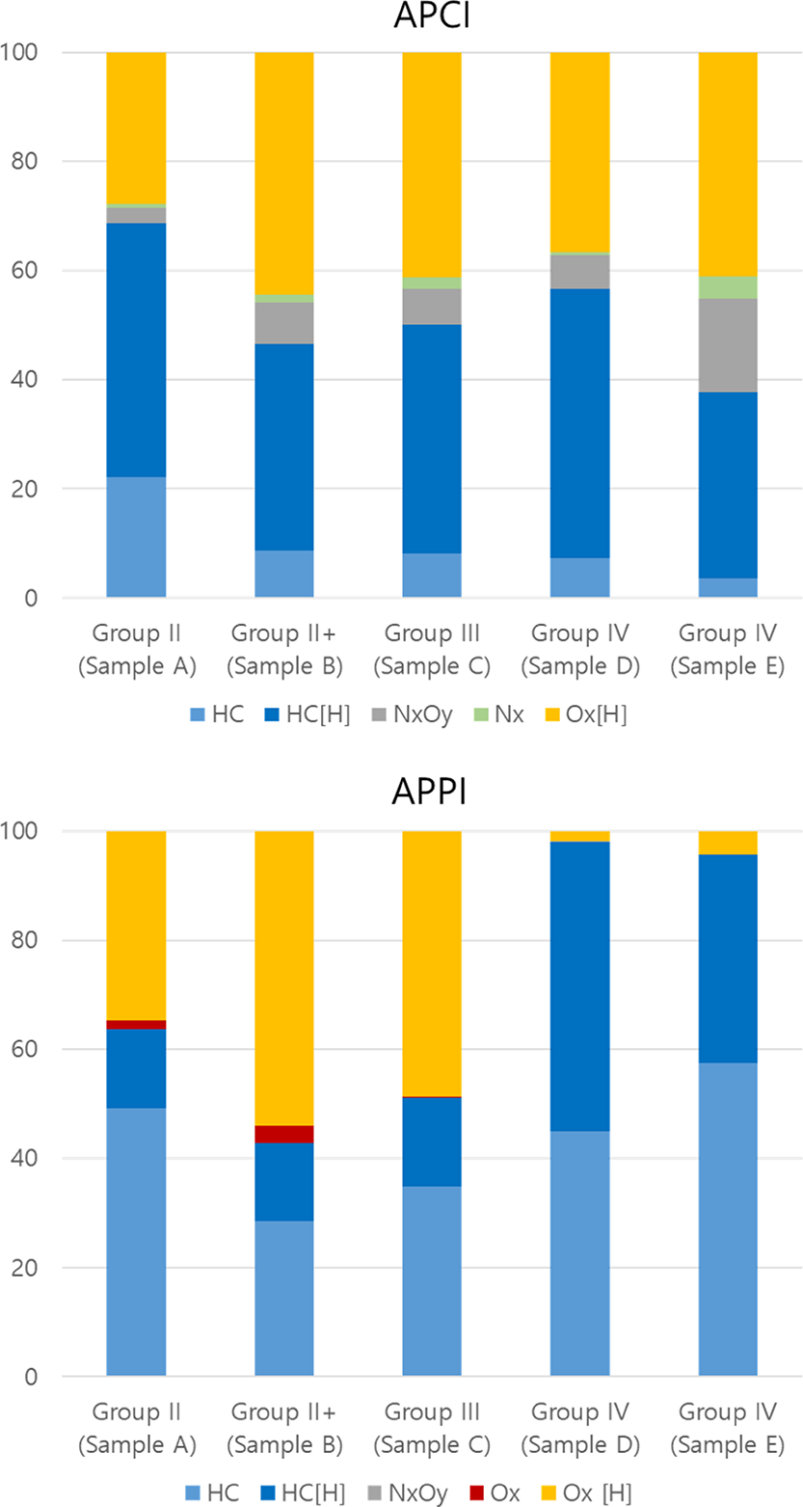
Class distributions of base oils by APCI/APPI FT-ICR MS.

To analyze the hydrocarbon compounds by FT-ICR MS, a suitable ionization method should be selected. The traditional ionization method applied for the analysis of linear alkanes is atmospheric pressure chemical ionization.^13^ However, soft ionization methods are very important to ensure the identification of molecular ions.^14,12^ In this study, the molecular composition of the hydrocarbons detected by both APCI and APPI is compared. The ultrahigh resolution achieved by FT-ICR MS allows a unique molecular formula to be assigned to each individual molecular composition within the mass spectra. Approximately 1800 and 1000 elemental compositions were detected in the mineral base oils and PAOs, respectively. The elemental compositions were classified by the heteroatomic class and presented in Figure 3 as a class distribution plot, where the classes including “[H]” in their names represent even-electron ions and classes not including this are odd-electron ions (radical ions).

**Figure 4:**
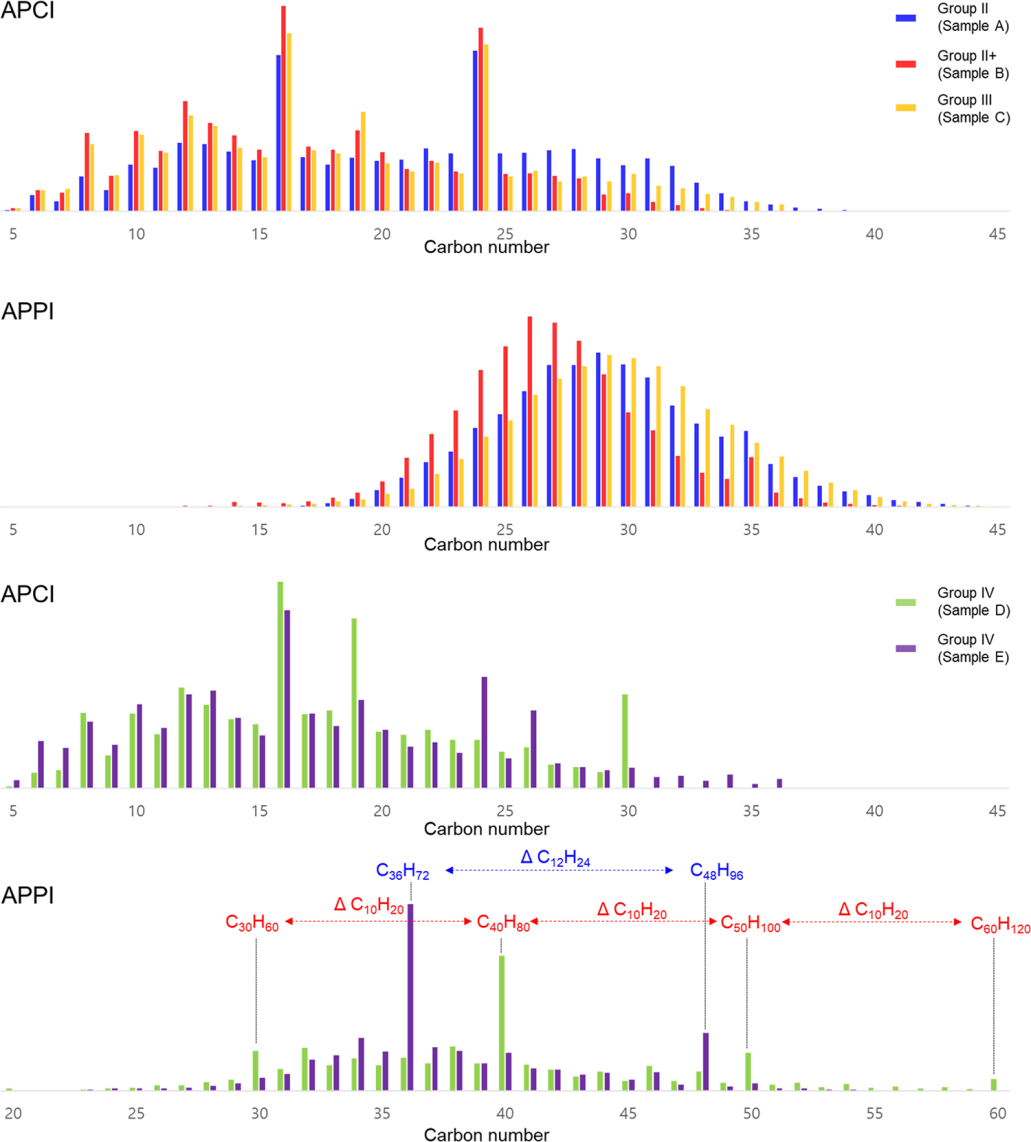
Carbon number distributions of base oils by APCI/APPI FT-ICR MS.

The main heteroatomic class detected by APCI corresponded to hydrocarbons (HC class), followed by oxygenated compounds (O*_x_*) and species containing nitrogen (N*_x_*). Similarly, HC compositions were detected with the highest intensity by APPI. However, nitrogen oxide (N*_x_*O*_y_*) compounds were hardly detected, and O*_x_* species were detected in lower relative abundance in comparison with APCI, in particular for Group IV (sample D/E) base oils. As a consequence of the combined effect of the high voltages applied to the corona needle and the presence of gases, solvents, and water vapor within the APCI source, an uptake of nitrogen and of oxygen, or of both, by the analyte is observed typically.^15^ Therefore, the N*_x_*, O*_x_*, or N*_x_*O*_y_* classes are likely ionization artifacts. The fragmentation trends for both ionization methods could also be seen in the carbon number distributions. Figure 4 shows the carbon number distributions of base oils using both ionization methods.

The compositions detected by APCI for Group II/II+/III base oils (samples A/B/C) were detected from carbon number 5 to 40. In comparison, higher molecular weight compounds were detected by APPI, from carbon number 15 to 44. Additionally, the compositions detected by APPI of mineral oils presented a homogeneous molecular distribution centered at C_29_ for Groups II and III and centered at C_26_ for Group II+. The shift in the carbon number distribution for Group II+, in comparison with other mineral oils, suggests that this oil may comprise shorter chain hydrocarbons.

Group IV base oils (samples D and E) also showed similar fragmentation tendencies to the mineral oils when ionized by APCI. Group IV compositions detected when using APPI were at a higher carbon number than those detected by APCI. Thus, a higher degree of fragmentation was observed by APCI. Group IV base oils are PAOs which form polymers by continuously connecting alpha-olefin monomers. Therefore, the identification of the alpha-olefin repeat unit is important to formerly characterize the PAO base oil.^13,16^

As can be seen in Figure 4, APPI can potentially determine the alpha-olefin monomer size. For instance, in sample D, a characteristic higher relative intensity was observed for peaks corresponding to the molecular compositions C_30_H_60_, C_40_H_80_, C_50_H_100_, and C_60_H_120_. The mass difference of these peaks corresponds to ΔC_10_H_20_. In sample E, the peaks with the highest relative intensity corresponded to C_36_H_72_, and C_48_H_96_ peaks correspond to a ΔC_12_H_24_ difference. This difference then corresponds to the size of the alpha-olefin repeat unit used to produce the PAO base oil. Thus, sample D is likely produced from 1-decene monomer and sample E from 1-dodecane monomer.

**Figure 5:**
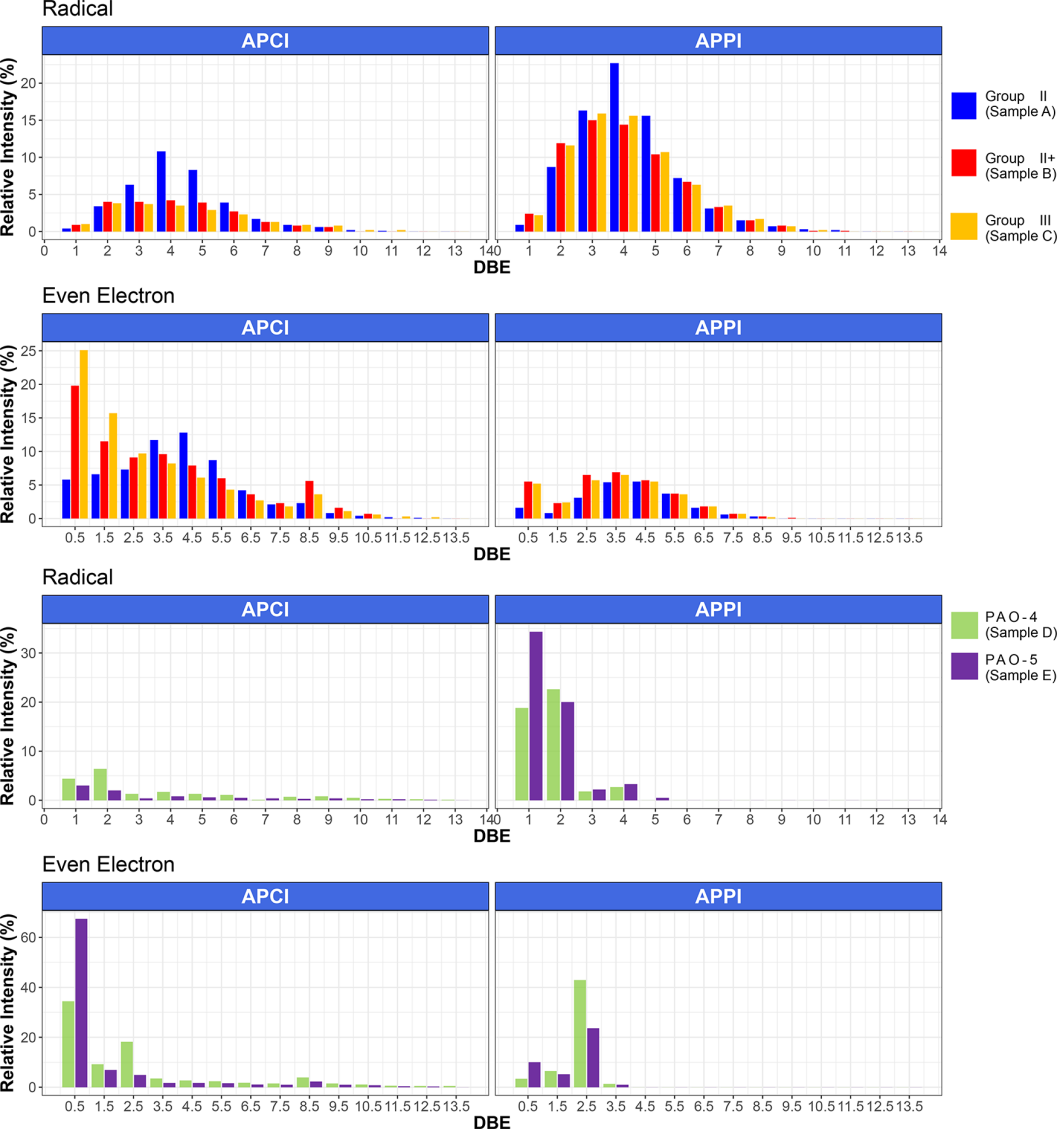
DBE distributions of base oils by APCI/APPI FT-ICR MS.

To investigate the difference in the chemical structures of the base oil components, a double-bond equivalent (DBE) plot was used. The DBE value of a molecule or ion indicates the total number of rings plus double bonds involving carbon atoms, and it can be calculated from the molecular formula (see eq 1); the DBE value also indicates the degree of unsaturation of a molecule.^17^ The DBE plot of the base oils using both ionization methods is shown in Figure 5.

According to the APCI results, the content of the compound with DBE 0.5 was observed with a high relative abundance in all base oils. As reported in the literature,^13^ the APCI ions are typically detected as [M – H]^+^ ions. Therefore, an even-electron species of DBE 0.5 corresponds to an alkane without a double bond in the neutral state. In particular, it was observed that the DBE 0.5 compounds of Group IV were detected with a relative abundance of 34% for sample D and 65% for sample E. In Group II/II+/III (samples A/B/C) base oils, linear hydrocarbon compounds with DBE 0.5 were detected with the highest intensity along with some polycyclic–alkane compounds with DBE > 0.5. On the other hand, cycloalkanes with DBE 2 and 3 were the main compounds detected by APPI. According to API definitions, Group II and III base oils should have a saturated hydrocarbon ratio of 90% or more.^18^ The presence of cycloalkanes, as detected by APPI, required further studies. Previous literature shows that cycloalkanes were also detected by 2D GC/MS at a high relative abundance.^19^

Similarly, Group IV base oils also presented linear alkanes with the highest relative abundance by APPI. In the case of analysis using GC–MS, only the information of aliphatic hydrocarbons was determined from the fragmentation. However, it was possible to obtain structural information for various compounds. APPI was a more suitable ionization method for analyzing the chemical structure of aliphatic hydrocarbons for base oils than APCI because APPI offered reduced fragmentation and reduced formation of artifacts, and therefore APPI represents better the original molecules present in the samples during ionization.

### 3.3 Combination of GC–MS and FT-ICR MS

When the GC–MS and FT-ICR MS data were used together, new information was obtained. According to previous literature, Group IV base oils are classified as PAOs and m-PAOs (m for metallocene) according to the chemical structure.^20^ PAO base oils present a nonuniform branch chain length, while m-PAO base oils present a regular branch orientation with uniform structure and longer chains. From the fragmentation patterns using the GC–MS data, sample D was found to have the alkyl branch length varying from C_16_H_33_ (theoretical: *m/z* 225.257676) to C_28_H_57_ (theoretical: *m/z* 393.445476). Using FT-ICR data, it was determined that the monomer unit was C_10_H_20_. If the molecular weight corresponding to the oligomer is subtracted from the molecular weight corresponding to the branched position, the length of the chain bonded in the form of a branch can be confirmed. The length of the branched chain for sample D was from C_2_H_4_ to C_8_H_17_, which was shorter than that of an oligomer. (Calculation formula example: C_22_H_44_–(C_10_H_20_*x* 2)) For sample E, the alkyl branch length was around C_22_H_45_ (theoretical: *m*/*z* 309.351576) from the GC–MS fragmentation data, while the oligomer size for sample E was determined to be C_12_H_24_ from the FT-ICR MS data. The length of the branched chain for sample E was around C_10_H_21_, which was similar to that of an oligomer (C_12_H_24_). Our results show that sample D is a PAO-type base oil with different lengths of alkyl chain branches, and sample E is more likely an m-PAO-type base oil with similar lengths of alkyl chain branches.

## 4 Conclusions

GC–MS and FT-ICR MS were used to characterize mineral and synthetic base oils. The soft ionization of the alkane constituents of base oils continues to be challenging. Both conventional EI (70 eV) and APCI yielded significant fragmentation, while the ions produced by APPI indicated higher DBE values than that expected for base oils. Both APPI and APCI also showed an increased abundance of oxygenated species due to ion–molecule reactions inside the respective ion sources. Nevertheless, the combination of data obtained from each ionization method can provide compositional information for base oils. GC–MS using EI was selective for the chemical structure and branched position of aliphatic hydrocarbons. APCI and APPI coupled to FT-ICR MS were used to determine the molecular formula of the base oils and of the PAO oligomers by ultrahigh resolution and mass accuracy. In turn, the molecular formula can be used to determine the DBE values and yield chemical structural information, such as alkanes, alkenes, and aromatics. When selecting an appropriate ionization method to reduce fragmentation and artifact formation, it is possible to determine the repeat unit for a PAO, which, ultimately, leads to structural characterization. The use of APPI led to less fragmentation for aliphatic hydrocarbons than when using APCI, reducing information loss. In addition, when using two complementary MS methods, it was possible to determine the degree of uniformity of the oligomers and alkyl branches, thereby obtaining information on the type of PAO.
